# TF-centered downstream gene set enrichment analysis: Inference of causal regulators by integrating TF-DNA interactions and protein post-translational modifications information

**DOI:** 10.1186/1471-2105-11-S11-S5

**Published:** 2010-12-14

**Authors:** Qi Liu, Yejun Tan, Tao Huang, Guohui Ding, Zhidong Tu, Lei Liu, Yixue Li, Hongyue Dai, Lu Xie

**Affiliations:** 1 School of Life Sciences & Biotechnology, Shanghai Jiao Tong University, Shanghai, 200240, China; 2 Shanghai Center for Bioinformation Technology, Shanghai, 200235, China; 3 Merck Research Laboratory, Rahway, NJ 07065, USA; 4 Merck Research Laboratory, Boston, MA 02115, USA

## Abstract

**Background:**

Inference of causal regulators responsible for gene expression changes under different conditions is of great importance but remains rather challenging. To date, most approaches use direct binding targets of transcription factors (TFs) to associate TFs with expression profiles. However, the low overlap between binding targets of a TF and the affected genes of the TF knockout limits the power of those methods.

**Results:**

We developed a TF-centered downstream gene set enrichment analysis approach to identify potential causal regulators responsible for expression changes. We constructed hierarchical and multi-layer regulation models to derive possible downstream gene sets of a TF using not only TF-DNA interactions, but also, for the first time, post-translational modifications (PTM) information. We verified our method in one expression dataset of large-scale TF knockout and another dataset involving both TF knockout and TF overexpression. Compared with the flat model using TF-DNA interactions alone, our method correctly identified five more actual perturbed TFs in large-scale TF knockout data and six more perturbed TFs in overexpression data. Potential regulatory pathways downstream of three perturbed regulators— SNF1, AFT1 and SUT1 —were given to demonstrate the power of multilayer regulation models integrating TF-DNA interactions and PTM information. Additionally, our method successfully identified known important TFs and inferred some novel potential TFs involved in the transition from fermentative to glycerol-based respiratory growth and in the pheromone response. Downstream regulation pathways of SUT1 and AFT1 were also supported by the mRNA and/or phosphorylation changes of their mediating TFs and/or “modulator” proteins.

**Conclusions:**

The results suggest that in addition to direct transcription, indirect transcription and post-translational regulation are also responsible for the effects of TFs perturbation, especially for TFs overexpression. Many TFs inferred by our method are supported by literature. Multiple TF regulation models could lead to new hypotheses for future experiments. Our method provides a valuable framework for analyzing gene expression data to identify causal regulators in the context of TF-DNA interactions and PTM information.

## Background

With the advance of high-throughput technologies such as DNA microarray, chromatin immunoprecipitation DNA chip (ChIP-chip) [1-3], yeast two-hybrid assays [4] and co-immunoprecipitation screens [5], various kinds of whole genome scale data are available, shedding light on the regulatory mechanisms in the biological system. Several new computational methods have been developed to combine various kinds of data to construct regulatory networks [6-11]. In addition, several researchers have strived to infer regulatory pathways connecting the known causal perturbation to the affected genes using physical interaction networks [12-15]. These inferred pathways could explain consequences of perturbations such as gene knockout effects. If the causal factor is unknown, however, inference of the causal factor from the consequences (e.g. a set of differentially expressed genes (DEGs)) is rather challenging.

To address this, Tu et al. [16] and Sutras et al. [17] integrated TF-DNA interactions and protein-protein interactions to map which gene among expression quantitative trait loci (eQTL) was the causal factor responsible for the observed changes in the downstream gene expression. However, the candidate causal factor was restricted to genes located within eQTLs, and their methods could not be widely applied if such information was not available. In another work, Pollard et al. [18] tried to discover underlying molecular causes of type 2 diabetes mellitus consistent with the expression changes based on 210,000 molecular cause-and-effect relationships assembled from literature. Yet the power of such kind of approach relies greatly on the size and quality of cause-and-effect relationships, which are often hard to collect.

Increasing amount of molecular interactions, including TF-DNA interactions, protein-protein interactions (PPI) and protein post-translational modifications (PTM), mapped from high-throughput technologies may provide significant information about cause-and-effect relationships. Previous approaches of associating TFs with expression changes were often based on direct binding targets of TFs [19-24], which were derived either by upstream sequence matches to a consensus binding motif [19-21,23], or by TF-DNA interactions from ChIP-chip experiments [21,22,24]. Several studies, however, have pointed out the low overlap between direct targets bound by a TF and transcriptionally affected genes caused by perturbation to the same TF [25-28]. Backup in regulatory pathways is one possible reason for the low overlap, which leads to no expression changes observed for most direct targets of a TF under this TF knockout [28]. The ability of TFs to affect gene expression through ways other than direct transcription may be another reason. Given the complexity of regulatory networks, if only TF-DNA interactions were used and simple flat regulation pathway was modeled, the power of those methods for inference of associated TFs would be limited. Integrating TF-DNA interactions with other directed interactions and considering hierarchical and multi-layer regulatory pathways through which TFs affect expressions of their downstream genes may be helpful. Protein-protein interactions provide limited information because PPIs normally imply no regulation direction. Protein post-translational modifications have rarely been considered for gene expression based causal inference, since PTM usually can not be detected at expression level.

Here we present a TF-centered downstream gene set enrichment analysis to identify potential causal regulators responsible for gene expression changes. Integrating TF-DNA interactions and PTM information, we constructed multi-layer regulation models progressively to derive possible downstream gene sets of a specific TF. PTM are incorporated because their regulation roles to proteins activation status are certain. TFs activity change would cause differential expressions of downstream genes, even though TFs themselves do not change at expression level. Compared with the method using only direct TF-DNA interactions, our method correctly identified five more actual perturbed TFs in knockout experiments [27] and six more TFs in overexpression experiments [29]. The results suggest that in addition to direct transcription, indirect transcription and PTM are also responsible for the downstream effects of TFs perturbation, especially for TFs overexpression. Potential regulatory pathways downstream of three perturbed regulators — SNF1, AFT1 and SUT1 — were given to demonstrate the power of incorporating indirect transcription and/or PTM information.

Furthermore, our method successfully identified several known and novel potential regulators involved in the transition from fermentative to glycerol-based respiratory growth [30] and in the pheromone response [31], some of which were validated by their changes in expression and/or phosphorylation status. Additionally, downstream regulation pathways of SUT1 in the transition process and AFT1 in the pheromone response were also supported by the mRNA and/or phosphorylation changes of their mediating TFs and/or “modulator” proteins.

Our results suggest that pathways through which TFs regulate the expression of downstream genes are condition dependent. With our methodology, many novel potential causal regulators and downstream regulation models may be proposed and evaluated. Our method provides a valuable framework for analyzing gene expression data to identify causal regulators in the context of TF-DNA interactions and PTM information, which may benefit disease mechanism studies and identification of potential interfering targets.

## Methods

**Figure 1:**
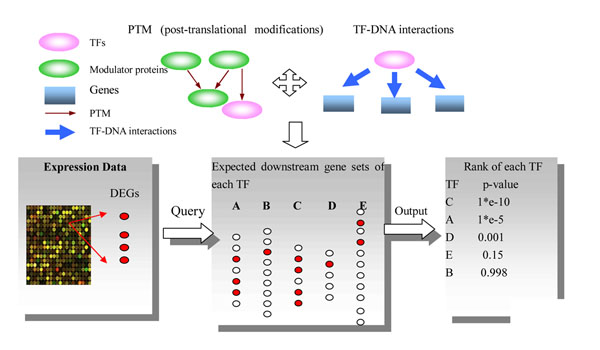
**The schema of our method.** The method ranked the potential causal regulators based on the overlap significance between downstream gene sets of each TF and the changed expression profiles, namely, the list of DEGs. The downstream gene sets of each TF were derived by integrating TF-DNA interactions and post-translational modifications (PTM) information.

The schema of our method is shown in Figure 1. For a perturbed condition, the method ranked the potential causal regulators based on the significance of the overlap between downstream gene sets of each TF and the changed expression profiles, namely, the list of DEGs. Compared with most previous approaches using direct TF-DNA interactions alone [19-24], the downstream gene sets of each TF here were derived by integrating TF-DNA interactions and PTM information.

**Figure 2:**
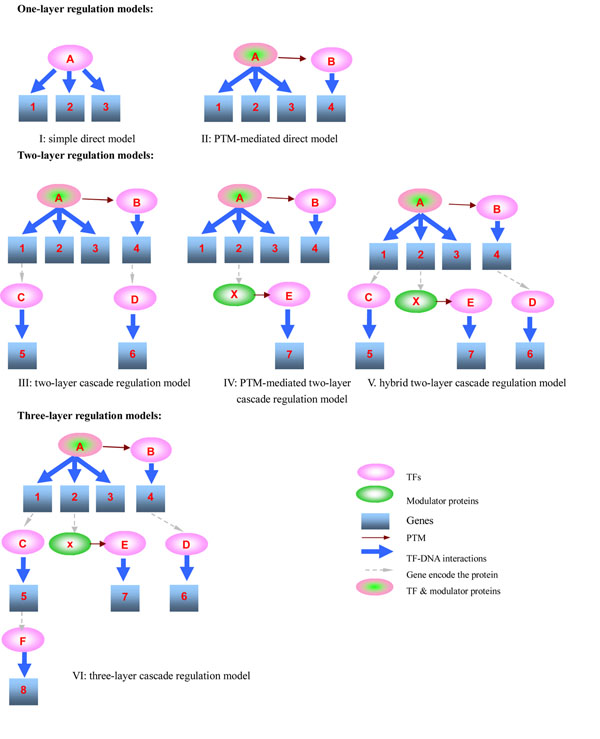
**Six models of downstream gene sets of TF A** I. simple direct model, downstream gene set of TF A: {1,2,3} II. PTM-mediated direct model, downstream gene set of TF A: {1,2,3,4} III. two-layer cascade regulation model, downstream gene set of TF A: {1,2,3,4,5,6} IV. PTM-mediated two-layer cascade regulation model: downstream gene set of TF A: {1,2,3,4,7} V. hybrid two-layer cascade regulation model, downstream gene set of TF A: {1,2,3,4,5,6,7} VI. three-layer cascade regulation model, downstream gene set of TF A: {1,2,3,4,5,6,7,8}

Recent studies have shown that gene perturbation effects could be mediated by PPI networks as well [12-14,16,17,28]. It has been suggested that when the path length from the initial TF to the last TF is greater than 2, the significance of the overlap between the observed DEGs and the expected targets of the actual perturbed TF is decreased [28]. Thus, we constructed regulation models with the path length from the initial TF to the last TF being equal to or less than 2. For examples, paths like TF-TF-TF, or TF-kinase-TF, are length 2 paths. Models were built progressively by gradually adding TF-DNA regulation layers and PTM information, as illustrated in Figure 2. Six models were constructed in total and classified into one-layer regulation models, two-layer regulation models and three-layer regulation models, which were defined according to the number of TF-DNA regulation layers. One-layer regulation models included simple direct model (flat model, Model I in Figure 2) and PTM-mediated direct model (Model II in Figure 2). Simple direct model (Model I) only considered direct binding targets of TF A, while PTM-mediated direct model (Model II) extended Model I to further include direct targets of other TFs (TF B in Figure 2) post-translationally modified by TF A. Model II could be further extended by another PTM layer, i.e., including direct targets of other TFs post-translationally modified by TF B. It had been found that downstream genes in this model were the same with those in model II for all TFs and only upstream kinases, such as ELM1, REG1, SAK1, GLC7 and TOS3, were involved in the model. Therefore this model was not included in our present work since the method was mainly aimed to identify TFs but not kinases responsible for expression changes. In the future, this model will be included when the work is extended to discover causal signal molecules and kinases. Two-layer regulation models consisted of two-layer cascade regulation model (Model III in Figure 2), PTM-mediated two-layer cascade regulation model (Model IV in Figure 2), and hybrid two-layer cascade regulation model (Model V in Figure 2). Two-layer cascade regulation model (Model III) was built by extending Model II to further include second-layer targets of TF A mediated by TF C and TF D, while PTM-mediated two-layer cascade regulation model (Model IV) was constructed by extending Model II to further include second-layer targets of TF A mediated by TF E which is modified by X (a ‘modulator’ and also a direct target of TF A). Hybrid two-layer cascade regulation model (Model V) combined Model III and Model I V, i.e., considering second-layer targets of TF A mediated by both protein expression changes (TF C and D) and protein modification changes (TF E). Three-layer regulation model (Model VI in Figure 2) was obtained by extending Model V to further include third-layer targets of TF A mediated by TF F. Model VI could not be further extended since the length of these paths TF A-TF C-TF F, TF A-‘modulator’ X-TF E, and TF A-TF B-TF D was all equal to 2. Thus there was only one three-layer regulation model.

Possible downstream genes of a TF were defined as the expected targets of this TF. Six possible expected targets for TF A were denoted as . The observed set of DEGs was denoted as D={DEGs}. The overlap significance between six expected targets and the observed set of DEGs was calculated using the hypergeometric distributions (equation 1).(1)

Six p-values were obtained for TF A to evaluate the significance of the overlap between six possible expected targets of TF A and the observed DEGs. To determine the most likely model through which TF A was responsible for expression changes, we selected the model in which the most significant overlap occurred (k* in equation 2). In this way, the overlap significance between the expected targets of TF A and the observed DEGs under this TF perturbation would be improved. However, the overlap significance between expected targets of other non-perturbed TFs and the DEGs might increase simultaneously, which would introduce much noise and descend the rank of the actual perturbed TF in the candidate list (Additional file S1). We assumed that if a TF functioned through a model rather than Model I, the overlap of its expected targets with the DEGs would be more significant in this model than that in Model I, while the overlap of most of other TFs in this model would not be more significant than that in Model I. On the contrary, if many TFs got more significant overlap between their expected targets and the observed DEGs in a model than that in Model I, these TFs would rank before the actual perturbed TF and thus the actual perturbed TF would be missed. That is, the model in which many TFs obtained more significant overlap than that in Model I was unacceptable. We compared the distribution of overlap p-values obtained by Model I and other models using the Wilcox rank sum test. Those models whose distribution of overlap p-values was significant different from Model I (p<=0.005) were unacceptable. The minimum p-value obtained at all acceptable models for each TF was used to rank and evaluate the TF as the potential causal regulators (equation 3). By selecting acceptable models based on the background distribution of overlap significance, we could correctly identify the actual perturbed TF and discover the most possible regulation pathway downstream of this TF perturbation (Additional file 1).(2)(3)

For the observed list of DEGs, we inferred the most likely causal regulators and the underlying pathways. P-values being equal to or less than 0.01 were considered to be significant and the corresponding TFs were reported as valid findings. If the actual perturbed TF was a valid finding and ranked at the top 20 of candidates, this TF was said to be correctly identified. We also tested our method using different numbers of top rank candidates to decide the correct identifications (top 15, top 25 and top 30 etc.). We found that the criterion about the number of top ranks had little impact on the performance of the method (Additional file 2), suggesting that the standard we used for the valid finding (p-value<=0.01) was quite stringent and most of valid findings ranked at the top. For previous methods that downstream genes of TFs were obtained through simple direct model (Model I) using TF-DNA interactions, only those TFs that affected gene expression through direct transcription could be correctly identified. In contrast, constructing hierarchical and multi-layer regulation models by the integration of TF-DNA interactions and PTM information, our method was expected to find not only TFs that affected gene expression through direct transcription, but also those that affected gene expression through indirect transcription and post-translational modifications.

We also built models in the following ways and compared the results from them with those from the six models: 1) We constructed models progressively without any constraint on the path length, i.e. with the path length from the initial TF to the last TF being greater than 2. We found that many TFs eventually got almost the same set of downstream genes when the path length was long enough and the method failed to infer most of the actual perturbed TFs. 2) We did not construct all six models fixedly but tried to infer which model explains the expression data best. For example, unlike direct targets of TF A, B, C and D comprising the downstream genes of TF A in the two-layer cascade regulation model (Model III), which combinations of TF B, C and D were inferred to comprise the regulation models of TF A in the case that regulation effects of TF A could be mediated by TF B, TF C and TF D. If direct targets of TF A and TF B obtained the most significant overlap with observed DEGs, this p-value was considered as the overlap significance obtained by TF A as causal regulators and used to rank the candidates. It was found that much more noise was introduced in this way and many actual perturbed TFs ranked lower in the candidate list. It is very likely that six models may not cover all the possible regulation topologies. However, we think some topologies happen at very low frequencies in real biological systems. If all the possible models were considered, it would increase the model space and lower the performance. Therefore, we limited our work to the six models with the belief that most frequent regulatory scenarios in the biological systems were well represented.

### Data collection

Post-translational modifications (PTM) information was obtained from *S. cerevisiae* phosphorylation network [32] and PTM-Switchboard [33]. The former contains the majority of the well-characterized kinase- and phophatase-substrate relationships in *S. cerevisiae* (654 and 141, respectively) manually curated from literature. The latter constitutes the relationship between the TF and its “modulator” protein, which alters the TF’s activity through post-translational modifications. TF-DNA interactions were obtained from [34], which presented a framework for integrating seven distinct sources of evidences to score all possible TF-target interactions. We extracted all TF-target interactions with LLS (Log Likelihood Score)>4, yielding a total of 13,239 high confidence interactions. By integrating PTM and TF-DNA interactions, 139 TFs with the number of downstream genes being no less than 4 in any one of the six models were selected as candidates (Names and the number of downstream genes of 139 TFs in each model are listed in Additional file 3).

Two TF perturbation data sets [27,29] were used to verify the method. Hu et al. [27] profiled the transcriptional response in *S. cerevisiae* strains with individual deletions of 269 TFs. Among 269 TFs, 128 are in aforementioned list of 139 candidates. The transcription responses of these 128 TFs knockout strains were selected to evaluate the power of the method. Chua et al. [29] provided the microarray expression data resulting from overexpression and/or deletion of 55 TFs, among which, those experiments of overexpression and/or deletion of TFs common with 139 candidates were selected. As a result, overexpression data of 39 TFs and deletion of 35 TFs were chosen. The standard p<=0.01 was used to select DEGs for Hu et al. data [27] and |z|>=2 was used for Chua et al. data [29] here. We also tested our method using different standards. We found that similar overlap p-values were obtained for most TFs even though different standards were used. Several TFs with very different overlap p-values often ranked at the top of the candidates. Furthermore, their ranks at the candidate list changed little, though their overlap p-values between the expected targets and the observed DEGs (chosen at different standards) changed a lot (detailed information in Additional file 2). Therefore, it may be concluded that our method is robust against the standard to select DEGs.

Our method was further applied to two datasets with no primary knockout or overexpression perturbation to discover important regulatory TFs involved in certain biological processes. Expression profiles during a transition from fermentative to glycerol-based respiratory growth were obtained from [30]. Expression data and phosphorylation information under pheromone response were from [31] and [52].

## Results and discussion

### Method verification by the knockout data of 128 TFs

**Figure 3:**
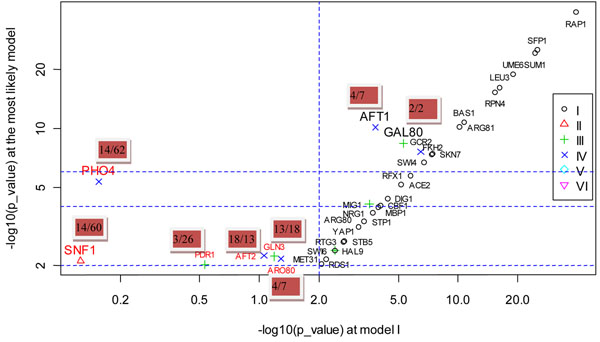
**The overlap significance obtained from the most likely model vs. that obtained from simple direct model on Hu et al.’s data.** note: different symbols denote the type of models for which the most overlap significance occurs. I: simple direct model; II: PTM-mediated direct model; III: two-layer cascade regulation model; IV:PTM-mediated two-layer cascade regulation model; V:hybrid two-layer cascade regulation model; VI:three-layer cascade regulation model. TFs with red fonts denote those correctly identified by our method but missed if only Model I was used. TFs with large size of fonts denote those whose overlaps between the expected and observed DEGs in the most likely model are 100 times more significant than that in Model I. The number in the red rectangle denotes the rank of the perturbed TF in the list of candidates (the rank obtained by our method/ the rank obtained if only Model I was used.).

**Figure 4:**
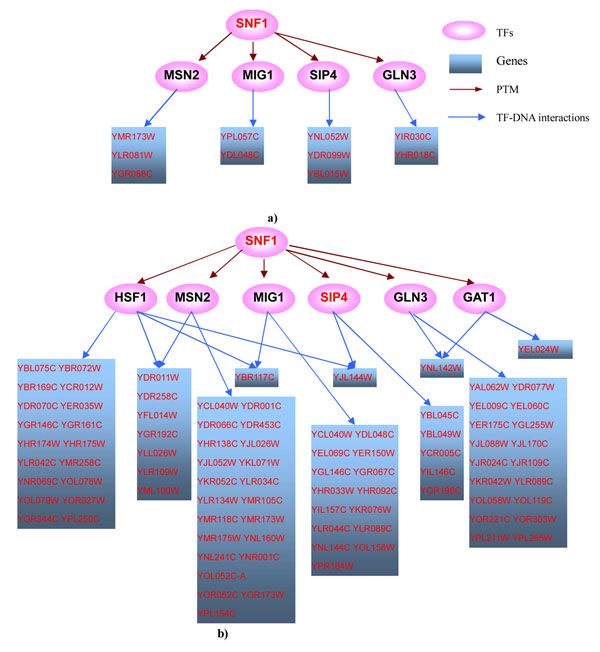
**a) Possible downstream regulatory pathway of SNF1 knockout. b) Possible downstream regulatory pathway of SNF1 in MAS1 mutant experiment.** note: TFs and genes with red fonts denote they are differentially expressed.

**Figure 5:**
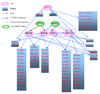
**Possible downstream regulatory pathway of AFT1 knockout**. note: TFs and genes with red fonts denote they are differentially expressed.

The results showed that 36 actual perturbed TFs out of 128 knockout data were correctly inferred. If only simple direct model (Model I) was considered, 31 were correctly inferred, among which, HSF1 was missed by our method due to its slightly dropped rank beyond the threshold. HSF1 ranked 20^th^ if only Model I was used but ranked 22^nd^ by our method. It may be due to the increased noise level introduced by considering more regulation models. Figure 3 shows the overlap significance of the expected targets of 36 TFs and the observed DEGs under these TFs’ knockout, obtained from the most likely model versus that obtained from Model I. It could be seen from the figure that Model I was employed by most TFs, suggesting that deletion of these TFs strongly affected expressions of their immediate direct targets. Six TFs — PHO4, GLN3, AFT2, SNF1, ARO80 and PDR1 — were correctly identified by our method but missed if only Model I was used. All of the TFs with the exception of AFT2 got more significant overlap in the most likely model, and their ranks in the candidate list were also improved. For example, PHO4 ranked 64^th^ if only Model I was used, while it ranked 14^th^ if Model IV was also considered. As another example, PDR1 ranked 26^th^ by using Model I alone, but ranked 3^rd^ if Model III was also considered. The more significant overlap and the improved ranks in the most likely model, rather than Model I alone, suggested that the effects of these TFs knockout might transmit through more complex models. Especially for PHO4 and SNF1, the overlaps between the downstream genes from the most likely model (PHO4 from Model IV, SNF1 from Model II) and the DEGs under their knockouts were 100 times more significant than that from Model I. This finding suggested that it was highly likely that the regulation pathway of PHO4 knockout was PTM-mediated two-layer cascade regulation model and pathway of SNF1 knockout was highly likely to be PTM-mediated direct model. Another two TFs, AFT1 and GAL80, were both correctly identified by our method and by Model I. However, their overlaps between the expected targets from the most likely model and the observed DEGs under their knockout were also much more significant than that from Model I and their ranks were also improved, suggesting the potential regulation pathways of AFT1 (Model IV) and GAL80 (Model III) knockout. These results demonstrated that PTM and cascade regulation helped explain TFs’ knockout effects. However, Hu et al. [27] did not observe any indication that indirect transcriptional or post-transcriptional regulation was responsible for the effects of a TF deletion. They drew the conclusion by comparing TF deletion target sets against each other or by comparing binding targets against deletion ones. This led to bias since the effects of a TF deletion might not be mediated by only one TF, but by cooperation or combination of several TFs. For example, the effects of SNF1 deletion are mediated by MSN2, MIG1, SIP4 and GLN3 (Figure 4). In another example, the effects of AFT1 deletion are mediated by cooperation of several TFs including MSN2, MSN4, DIG1 and STE12 (Figure 5).

In higher eukaryotes, multiple TFs simultaneously, cooperatively or competitively, regulate genes. For those TFs forming transcription complex to coordinate the expression of genes, such as ARG80/ARG81, SWI4/SWI6 and MBP1/SWI6, they were all identified as causal regulators when one of their cooperative TFs was knocked out. For examples, ARG81 ranked 1^st^ (p-value=6.61e-11) and ARG80 ranked 6^th^ (p-value=2.12e-5) in the ARG81 knockout experiment, and ARG81 ranked 1^st^ (p-value=9.81e-9) and ARG80 ranked 19^th^ (p-value=6.97e-4) in the ARG80 knockout experiment. For those partially functionally redundant TFs (e.g., MSN2/MSN4) or promoter occupancy competitive TFs (e.g., YRR1/YRM1), however, they could not be correctly identified as causal regulators in their knockout experiments. It is possibly because that those TFs’ knockout effects can be partially or fully compensated by their backup TFs or competitive TFs.

The results (including the number of downstream genes of every TF in each model, the number of DEGs, the number and the p-value of the overlap between downstream genes and DEGs in each model, the minimum p-value obtained from six models and the minimum p-value obtained from acceptable models) of the knockout data of 128 TFs are listed in Additional file 4.

#### Possible regulatory pathways downstream of SNF1 knockout

SNF1 achieved the most significant overlap p-value (0.0077) from PTM-mediated direct model (Model II), while the overlap p-value was 1 (the number of the overlap between the observed DEGs of SNF1 knockout and the expected targets was 0) when only simple direct model was used. This may suggest that SNF1 affects gene expression through a PTM-mediated way, i.e., SNF1 functions as a kinase rather than a TF. This finding is consistent with previous literature. The SNF1p kinase complex, which phosphorylates serine and threonine residues, is essential for regulating the transcriptional changes associated with glucose depression through its deactivation of the transcriptional repressor MIG1 [35]. SNF1 is involved in the activation of *S. cerevisiae* heat shock transcription factor under glucose starvation conditions [36]. SNF1p is also known or predicted to phosphorylate a wide range of substrates, including SIP4 [37], MSN2 [38], GAT1 and GLN3 [39].

To further verify whether or not SNF1 functions through a PTM-mediated way, we selected those expression experiments in which SNF1 was up/down-regulated [40] assuming that differential expression of SNF1 would lead to expression changes of its downstream genes, thus SNF1 could be inferred as the causal regulator. We compared the overlap significance between the DEGs of those data and the expected targets of SNF1 in the six models. When PTM-mediated direct model was used, the overlaps between the observed DEGs and the expected targets of SNF1 were highly significant (Table 1, p-value<=0.001), while the number of the overlap was 0 (p-value=1) when other models were used. This further supports that SNF1 functions as a kinase. Figure 4 shows possible regulatory mechanisms downstream of SNF1 knockout and in MAS1 promoter mutant experiments [40]. Though the overlap of DEGs between these two conditions is low, gene expressions are affected through similar pathways. SNF1 regulates gene expressions through its modification of HSF1, MSN2, MIG1, SIP4, GAT1 and GLN3 in the MAS1 promoter mutant experiment, while SNF1 affects expressions through MSN2, MIG1, SIP4 and GLN3 in the knockout experiment. The low overlap may be due to the involvement of other TFs unrelated to SNF1.

**Table 1:** Differential expression of SNF1 and the overlap p-values of the observed DEGs and the expected downstream genes using PTM-mediated direct model (Model II)

Experiments	Fold change	Number of DEGs	p-value at Model II
ARC40 promoter mutant	-3.3	1887	7.77931e-4

RPT2 promoter mutant	3.2	879	3.43575e-4

MAS1 promoter mutant	-3.3	500	3.11418e-10

GPI2 promoter mutant	3.2	319	2.0351e-4

ESF1 promoter mutant	3.2	392	2.1737e-06

#### Possible regulatory pathways downstream of AFT1 knockout

When simple direct model was used, the overlap p-value between the expected targets of AFT1 and the observed DEGs under AFT1 knockout was 0.0001, while the overlap p-value was 7.35e-11 when PTM-mediated two-layer cascade regulation model (Model IV) was used. Figure 5 illustrates PTM-mediated two-layer cascade regulation pathway downstream of AFT1 knockout. AFT1 affects the expression of two protein kinases, TPK1 and KSS1. TPK1 modifies the protein states of MSN2 and MSN4, while KSS1 modifies the protein states of DIG1 and STE12. Then these TFs lead to differential expressions of their target genes. TPK1 was differentially expressed in the AFT1 knockout experiment, which supported the possibility of this regulatory pathway. The expression of KSS1 was not changed, possibly due to the feedback regulation control of DIG1, STE12 and MSN4 on KSS1.

To further verify whether or not AFT1 functions through Model IV under specific conditions, expression data with great down expression of AFT1 were selected [41]. In the response to environmental changes under YPD stationary phase 28 d, AFT1 showed 9.6 fold change of down expression. We compared the DEGs under this condition with the expected targets in the six models of AFT1, the most significant overlap was also achieved at PTM-mediated two-layer cascade regulation model (p-value:e-17).

### Method verification by the overexpression of 39 TFs and deletion of 35 TFs

11 Actual perturbed TFs out of 35 TFs deletion experiments were correctly identified, while 17 perturbed TFs out of 39 TFs overexpression data were correctly identified. In comparison, 11 perturbed TFs out of 35 TFs deletion experiments and 11 out of 39 TFs overexpression ones were correctly inferred if only Model I was used. HAP4 and ROX1 were correctly identified by Model I but missed by our method in their knockout experiments. Although the same p-values were obtained (0.002675), HAP4 ranked 4^th^ if Model I was used but ranked 22^nd^ by our method. The dropped rank of HAP4 may be partially due to the increased noise level introduced by considering more models in our method. Additionally, considering no cooperative or competitive interactions between TFs may be another reason. In the HAP4 knockout experiment, HAP5 forming HAP complex with HAP4 ranked 1^st^, though HAP4 itself ranked 22^nd^. If such cooperative interaction between HAP5 and HAP4 was integrated into the method, HAP4 would rank much higher. The situation was similar for ROX1, which ranked 4^th^ if Model I was used and ranked 29^th^ by our method. Our method correctly identified SOK2 and SUT1, which were missed by Model I in deletion experiments. Our method also correctly identified six more perturbed TFs (MIG1, YAP1, INO2, MBP1, XBP1 and SUT1) from their corresponding overexpression experiments. The result showed that considering PTM and cascade regulation helped explain effects of TFs perturbation, especially effects of TFs overexpression. The results (including the number of expected targets of every TF in each model, the number of DEGs, the number and the significance of the overlap between the expected targets and the DEGs, the minimum p-value obtained from the six models and the minimum p-value from acceptable models) of the deletion of 35 TFs and overexpression of 39 TFs are listed in Additional file 5.

**Figure 6:**
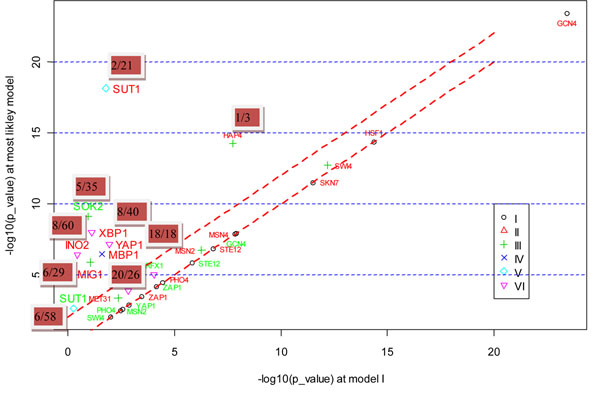
**The overlap significance obtained from the most likely models vs. that obtained from simple direct model on Chua et al.’s data.** note: different symbols denote the type of models for which the most overlap significance occurs. I: simple direct model; II: PTM-mediated direct model; III: two-layer cascade regulation model; IV: PTM-mediated two-layer cascade regulation model; V: hybrid two-layer cascade regulation model; VI:three-layer cascade regulation model. TFs with red fonts denote those correctly identified in overexpression experiments. TFs with green fonts denote those correctly identified in deletion experiments. TFs with large size of fonts denote those correctly identified in the most likely model but missed in Model I. The number in the red rectangle denotes the rank of the TF in the list of candidates (the rank obtained by our method/ the rank obtained if only Model I was used).

Figure 6 shows the overlap significance of 11 TFs and 19 TFs between the expected targets obtained from the most likely model and the observed DEGs in deletion and/or overexpression experiments versus that obtained using Model I only. In deletion experiments, the significance of the overlaps between the expected targets from their most likely models for SKO2 and SUT1 and the observed DEGs was not only much higher than that from Model I (100 times more significant), their ranks in the candidate list were also improved, which suggested that the effects of SOK2 deletion were mediated by Model III and the effects of SUT1 deletion were mediated by Model V. For overexpression experiments of six TFs, MIG1, YAP1, INO2, MBP1, XBP1 and SUT1, the overlaps between their expected targets from their most likely models and observed DEGs were much higher than that from Model I (100 times more significant), their ranks were also improved a lot. This indicated that the effects of those TFs’ overexpression were mediated by their most likely models other than Model I. HAP4 could be correctly identified by Model I, however, it achieved higher overlap significance between the expected targets from Model III and the observed DEGs under HAP4 overexpression, and it ranked 1^st^ in Model III. This indicated that the effects of HAP4 overexpression might be mediated through Model III.

With the exception of ABF1, HSF1, MET4 and RAP1, which appeared only in overexpression experiments, the remaining 32 TFs were performed with both overexpression and deletion experiments. 7 of 32 TFs (MSN2, MSN4, GCN4, SWI4, STE12, SKN7 and PHO4) showed higher overlap significance between the direct targets of these TFs and the observed DEGs in overexpression experiments than those in deletion ones. This is partially due to the redundancy of TFs. For example, MSN2 and MSN4 are paralogous TFs with BLASTP E-value less than E-20, SWI4 and SKN7 have paralogous TFs with BLASTP E-value less than E-10. A subset of the homologous TFs bind to an overlapping group of targets, and thus it is not surprising that knocking out one of them has a small effect on the expression of its targets. In contrast to deletion, overexpression increases binding of TFs, and thus would activate most of their downstream targets. 2 out of 11 TFs in deletion experiments (SUT1 and SOK2), and 6 out of 17 TFs in overexpression experiments showed more significant overlap between the DEGs and the expected targets in other models than in Model I by 100 times. This may suggest that effects of TFs overexpression are more involved in indirect transcriptional or post-transcriptional regulation than those of TFs deletion.

We also compared our results on deletion experiments from [27] and those from [29]. Simple direct model was responsible for the effects of two SWI4 deletion experiments. For some TFs, however, different pathways were involved in the downstream of two deletion experiments of the same TF. For example, simple direct transcription explained the effects of PHO4 deletion experiment provided by [29], while PTM-mediated two-layer cascade regulation model was the most likely model for the effects of the PHO4 deletion experiment provided by [27]. This may imply that indirect transcriptional or post-transcriptional regulatory pathways downstream of TFs are condition-dependent, even in the case of the same TF deletion.

It should be noted that despite our prediction rate was less than 50%, it was much higher than that by random guess. If we randomly selected 20 TFs from the 139 candidates as causal regulators, the prediction rate was 14% (20/139). It would be even much lower if the quite stringent standard we used about valid findings (p-value<=0.01) was also required since only small number of TFs satisfied the standard. In comparison, the prediction rate of our method achieved 28% (36/128) on Hu et al. data, 31% (11/35) on Chua et al. deletion data and 44% (17/39) on Chua et al. overexpression data.

#### Possible regulatory pathways downstream of SUT1 overexpression

**Figure 7:**
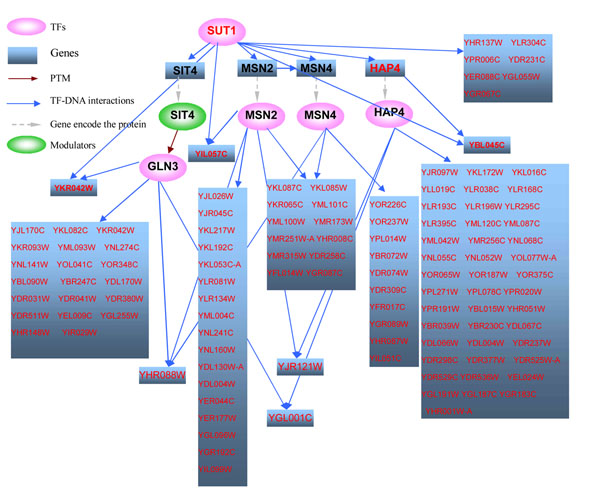
**Possible downstream regulatory pathway of SUT1 overexpression** Note: TFs and genes with red fonts denote they are differentially expressed

When simple direct model was used, the overlap p-value between the expected downstream genes of SUT1 and the observed DEGs under SUT1 overexpression was 0.017, while p-value was 7.37e-19 when hybrid two-layer cascade regulation model (Model V) was used, and SUT1 ranked 2^nd^ as the causal regulator out of 139 TF candidates. Figure 7 shows the possible regulatory pathway downstream of SUT1 overexpression. The effects of SUT1 overexpression are mediated by GLN3, MSN2, MSN4 and HAP4. Among them, activity of GLN3 is mediated by the phosphatase SIT4. SIT4, MSN2, MSN4 and HAP4 are all direct targets of SUT1. Down expression was also observed for HAP4 when SUT1 was overexpressed, which further supported this regulatory pathway of SUT1.

### Method applied to transcriptome profiling during a transition from fermentative to glycerol-based respiratory growth

To demonstrate the utility of the method to identify activated TFs in real biological process, we applied the method on transcriptome profiling during a transition from fermentative to glycerol-based respiratory growth [30]. We constructed a gold positive set as true activated TFs by selecting those TFs with two-fold or more differential expression from 139 candidates. The gold positive set consisted of 33 TFs.

Our method identified 55 TFs with overlap p-value being equal to or less than 0.01. Among 55 TFs, 18 TFs were true positives (TP, i.e., their expression were up-regulated or down-regulated two-fold or more), expression of 35 TFs were changed less than two-fold (i.e., these 35 TFs were false positives, FP), MAL63 and MATA2 were not profiled by the microarray. 15 TFs of the gold positive set were not detected by our method (false negatives, FN). In comparison, only 14 TFs with overlap significance p-value being equal to or less than 0.01 were inferred if only simple direct model was used. Among them, 6 TFs were two-fold or more differentially regulated (TP), 8 TFs were less than two-fold differentially expressed (FP). 27 TFs of the gold positive set were missed (FN). We compared the Jaccard Similarity Score of our results with that obtained from simple direct model (Table 2). The Jaccard Similarity Score was defined as TP/(TP+FP+FN) [8]. The higher the Jaccard Similarity Score, the better the method performs. The result showed that although our method got higher false positives, it achieved higher Jaccard Similarity Score than that from Model I, suggesting that taking multilayer-wise regulation models and PTM information into consideration was helpful for identifying activated TFs in the biological process.

**Table 2:** Jaccard similarity score obtained by our method and by simple direct model during a transition from fermentative to glycerol-based respiratory growth

	TP	FP	FN	Jaccard similarity score
Our method	18	35	15	0.265

Direct model	6	8	27	0.146

The high false positives of our method may be due to the incomplete selection of positive set, where only those TFs with differential mRNA expression were chosen. This standard would miss those activated TFs with changed protein status (e.g. changed protein level or changed protein modifications status). Among the top 20 causal regulators identified by our method (Table 3), 15 regulators could not be inferred if only simple direct model was used (p-value>=0.01 in Model I). 4 of 15 regulators (SUT1, XBP1, AZF1 and HAP1) were differentially expressed, which suggested the possibility of their activity changes in the process. The remaining 11 regulators were treated as false positives. However, the involvement of these 11 regulators in the diauxic shift is known or can be inferred by literature. For example, SNF1 and SSN3 are both important regulators involved in the diauxic shift [42-45]. The SNF1 protein kinase controls the induction of genes of the iron uptake pathway at the diauxic shift [43]. In glucose-grown cells, SSN3 negatively regulates 173 genes, including 75 that are induced during the diauxic shift [44]. Moreover, SSN3 protein levels are depleted during the diauxic shift [44] and SSN3 is also required for phosphorylation of SIP4 during growth in nonfermentable carbon sources [45]. As another example, PHO4 is an in vivo substrate of PHO85, one of the yeast nutrient-sensing kinases involved in the changes in gene expression profiles when yeast cells undergo a diauxic shift [46]. Additionally, PHO4 functions to repress or down-regulate the transcription of SNZ1, which is expressed specifically in the postdiauxic to stationary phase [47]. In another example, CBF1 DNA binding is necessary for repression of PHO8 basal expression at the diauxic shift [48]. It has been reported that carbon deprivation caused the nuclear localization of YAP1 [49] and the proportion of cells with the nuclear YAP1 concentrated during the diauxic shift [50], which suggests that YAP1 is involved in the process. PUT3 is also predicted to be involved in the process [51]. These regulators are possibly true positives and are activated in the process via changes of protein status.

**Table 3:** The top 20 identified activated TFs during a transition from fermentative to glycerol-based respiratory growth

ORF	TF name	p-value at Model I	p-value at most likely model	Differentially expressed
YGL035C	MIG1	0.004279	3.72E-09	

YHR206W	SKN7	0.007001	5.73E-09	

YDR043C	NRG1	3.69E-06	1.81E-08	YES

YGL162W	SUT1	0.030191	1.87E-08	YES

YDR259C	YAP6	0.054747	7.44E-08	

YPR065W	ROX1	0.008967	1.38E-07	YES

YKL043W	PHD1	0.685377	2.97E-07	

YOR028C	CIN5	0.024372	4.84E-07	

YKL112W	ABF1	0.687202	5.10E-07	

YDR477W	SNF1	1	9.05E-07	

YIL101C	XBP1	0.19727	2.27E-06	YES

YOR113W	AZF1	0.068534	2.75E-06	YES

YML007W	YAP1	0.01445	6.76E-06	

YKL109W	HAP4	4.69E-05	1.09E-05	YES

YKL015W	PUT3	0.036939	1.15E-05	

YLR256W	HAP1	0.068331	1.41E-05	YES

YOR372C	NDD1	0.402404	2.37E-05	

YJR060W	CBF1	0.029986	2.54E-05	

YPL042C	SSN3	1	4.34E-05	

YFR034C	PHO4	0.503024	4.53E-05	

ROX1 and SUT1 ranked 6^th^ and 4^th^, respectively, which were both differentially expressed. SUT1 was the target of ROX1, and SUT1 affected the expression of genes through hybrid two-layer cascade regulation model (Model V). This was consistent with the pathway of SUT1 illustrated in Figure 7, where the perturbation of SUT1 was mediated by GLN3, MSN2, MSN4 and HAP4. SIT4 (mediating the activity of GLN3), MSN2 and HAP4 were differentially expressed in the transition process, which further supported the possibility of the regulatory pathway of SUT1.

The overlap p-values of each TF in six models and the minimum p-value of each TF are listed in Additional file 6. TFs are ranked as causal regulators by their minimum p-values.

### Results on the expression profiles in the transition from fermentative to glycerol-based respiratory growth.

Having demonstrated that our method identified those activated TFs with differential expression, we tested whether our method could find activated TFs that were both differentially expressed and post-translationally modified. We applied our method to infer activated TFs for mRNA profiling data sets for yeast responding to the mating pheromone factor (wild-type cells after treatment with 50 nM α-factor for 120 min) [31].Those identified TFs were evaluated by the transcriptional data and phosphoproteomics data after treatment with 2 mM α-factor for 120 min [52]. Although the treatment concentrations were different between these two datasets, there was evidence that the transcriptional response to α-factor saturated at concentrations above 15.8 nM [31].

Table 4 lists the top 10 identified TFs by our method. Among them, five TFs were differentially regulated and/or differentially phosphorylated, out of which, MSN4 would be missed if only simple direct model was used. SWI4 and STE12 were both differentially regulated and phosphorylated. Increased phosphorylation was observed for DIG1 (3.8 fold) and MSN4 (two fold). Increased mRNA expression was observed for TEC1. The involvement of the remaining five TFs (SWI6, MCM1, SSN3, SKN7 and AFT1) is known or can be inferred from literature. MCM1 in cooperation with STE12 (differentially regulated and phosphorylated) regulates cell cycle-dependent transcription of FAR1 (differentially regulated and phosphorylated) [53], which are essential for pheromone-induced cell cycle arrest. FAR1 is activated by MSN2 and repressed by the SSN3 kinase [54], indicating the possible involvement of SSN3 in the process. CDC28-CLN3 complex activates SBF (SWI4-SWI6) and MBF (MBP1-SWI4), and the function of CLN3 in G1 phase, including control of cell size and pheromone sensitivity, requires the protein of SWI6 [55]. Decreased phosphorylated sites were observed for SWI4 and CDC28. Therefore, it can be inferred that SWI6 is also involved in the process. To survive pheromone stress, the yeast *S. cerevisiae* activates signaling through the Ca2+-activated phosphatase calcineurin to the transcription factor Crz1p , SKN7 are necessary for Crz1p-dependent transcriptional activation and Crz1p stabilization by SKN7 in vivo[56], which suggests that SKN7 is also an important regulator in the process. For AFT1, significant overlap (p-value=e-18) was obtained when PTM-mediated two-layer cascade regulation model (Model IV) was used. We found that the regulation pathway of AFT1 ( Figure 5) was supported by mRNA expression and phosphorylation changes of mediating TFs and kinases, where TPK1 was up-regulated, and MSN4, DIG1 and STE12 were all observed for hyperphosphorylation. In addition, overexpression of AFT1 leads to growth arrest of the G1 state [57], while pheromone also induces arrest in G1 phase. AFT1 was also predicted to be involved in the pheromone response [58].

The overlap p-values of each TF in six models and the minimum p-value of each TF are listed in Additional file 7. TFs were ranked as causal regulators by their minimum p-values.

**Table 4:** The top 10 identified activated TFs in the pheromone response

TF name	p-value at Model I	p-value at the most likely model	Differentially expressed	Differentially phosphorylated
SWI6	7.13E-15	4.77E-29		

AFT1	0.153789	6.78E-29		

STE12	3.82E-20	1.02E-27	YES	YES

DIG1	5.27E-12	2.11E-24		YES

MSN4	0.04847	2.43E-23		YES

MCM1	2.25E-10	3.87E-22		

TEC1	7.84E-07	4.36E-21	YES	

SSN3	1	6.19E-21		

SWI4	1.01E-12	1.05E-20	YES	YES

SKN7	0.02109	1.15E-20		

## Conclusions

Our work provides an initial step toward analyzing gene expression data to find causal regulators by integrating TF-DNA interactions and PTM information. We tested our method on large-scale TF deletion and overexpression experiments. The method correctly identified more actual perturbed TFs than the approach using only direct transcription, suggesting that indirect transcription and post-translation regulation are also responsible for TFs’ deletion/overexpression effects, especially for TFs’ overexpression effects. Our method successfully identified known causal regulators and also inferred some novel TFs, which could lead to new hypotheses for future experiments during the processes of a transition from fermentative to glycerol-based respiratory growth and pheromone response. Furthermore, possible regulatory pathways downstream of TFs in these processes were presented. Expression and phosphorylation states of genes/proteins in the regulatory pathways provided further evidence to support the validity of these pathways.

Although our method was developed to find causal TFs, it could be easily extended to discover causal signal molecules and kinases. For example, CDC28 is a catalytic subunit of the main cell cycle cyclin-dependent kinase. If downstream gene set of CDC28 was constructed by PTM-mediated direct model, the number of the overlap between the observed DEGs (number:1072) in pheromone response and expected targets of CDC28 (number: 969) was 270 and the p-value was 1.229705e-18. This suggests that CDC28 is involved in pheromone response, a finding that is supported by literature and consistent with experiments showing reduced phosphorylation of CDC28 [52].

In future work, we can further improve the method in several ways: 1) It could be beneficial to integrate into the framework information of transcriptional complexes and cooperative and competitive interactions. For example, although GCR1 was not correctly identified, RAP1 was identified with the 2^nd^ rank (p-value: 5.53e-32) in GCR1 knockout experiment. It is known that the RAP1/GCR1 regulatory complex is required for efficient transcription of ribosomal protein (RP) and glycolytic genes [59]. If such information of the cooperative interaction between RAP1 and GCR1 was integrated into the framework, GCR1 could be correctly identified as the causal regulator. 2) It could be helpful to integrate into the framework proteomics data. Protein abundance together with protein post-translational modifications status could be used to calculate the probability of existence of pathways, and then evaluate the possibility of the regulator as the cause. 3) It may be helpful to add sign in the model to represent the regulatory effects (activation or repression), to enhance the preciseness of the framework. 4) Our method would miss those TFs only responsible for small subset of DEGs because overlaps between downstream genes of those TFs and the whole set of DEGs would not be significant. Constructing downstream genes of combination of TFs may be beneficial in these cases. 5) The performance of our method relies heavily the size and quality of TF-DNA interactions and PTM information. However, all of the interactions, especially PTM information, are incomplete and variable in different cellular conditions. Limited and inaccurate information could mislead to biased paths and causal regulators. More abundant and accurate data available in the future would improve the power of the method.

## Competing interests

'The authors declare that they have no competing interests.

## Authors' contributions

QL developed software, analyzed data and wrote the manuscript. HD, YL and LX designed the project direction. TH and GD participated in project discussions. YT, ZT, LL, YL, HD and LX supervised the study. YT ,ZT and LX revised the manuscript. All authors read and approved the final manuscript.

## Supplementary Material

Additional file 1 Rank distribution of the perturbed TF obtained by the method only using Model I, by the method selecting the minimum p-value from the six models and by the method selecting the minimum p-value from acceptable models. Click here for file

Additional file 2 the effects of parameters to determine proper TFs and select DEGs on the results. Click here for file

Additional file 3 139 TFs with the number of downstream genes being no less than 4 in any one of the six models. ORF names, gene names, and the number of downstream genes obtained by each model for 139 TFs are listed. Click here for file

Additional file 4 Results on knockout data of 128 TFs. Click here for file

Additional file 5 Results on deletion experiments of 35 TFs and overexpression ones of 39 TFs. Click here for file

Additional file 6 Results on the expression profiles in the transition from fermentative to glycerol-based respiratory growth. Click here for file

Additional file 7 Results on the expression data in the pheromone response. Click here for file
