# Supplementary material for: TF-centered downstream gene set enrichment analysis: Inference of causal regulators by integrating TF-DNA interactions and protein post-translational modifications information
Source: BMC Bioinformatics. 2010 Dec 14;11(Suppl 11):S5. doi: 10.1186/1471-2105-11-S11-S5 (PMC3024863; doi:10.1186/1471-2105-11-S11-S5)
Supplement: Additional file 1 — Rank distribution of the perturbed TF obtained by the method only using Model I, by the method selecting the minimum p-value from the six models and by the method selecting the minimum p-value from acceptable models. [file 1471-2105-11-S11-S5-S1.pdf]

Supplementary Figure S1

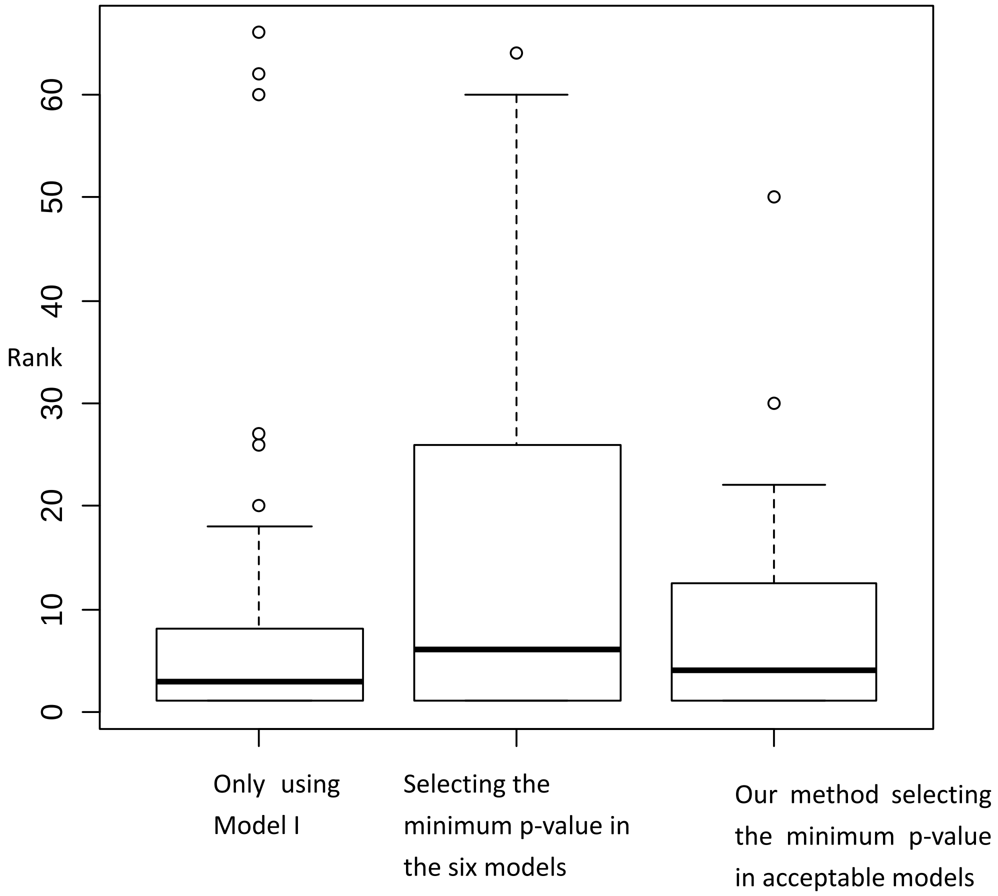

Figure s1: the rank distribution of the actual perturbed TF in the 139 candidates list. Although the method selecting the minimum p-value in the six models would lead to a more significant overlap between expected targets of the TF and the observed DEGs, but it dropped the rank of the actual perturbed TF in the candidates list due to the increased noise level introduced from more models considered. Our method tried to improve the overlap and not to drop the rank of the TF by selecting acceptable models. The figure shows that selecting acceptable models help to reduce the noise and sustain the rank of actual perturbed TF.
