# Supplementary material for: TF-centered downstream gene set enrichment analysis: Inference of causal regulators by integrating TF-DNA interactions and protein post-translational modifications information
Source: BMC Bioinformatics. 2010 Dec 14;11(Suppl 11):S5. doi: 10.1186/1471-2105-11-S11-S5 (PMC3024863; doi:10.1186/1471-2105-11-S11-S5)
Supplement: Additional file 2 — the effects of parameters to determine proper TFs and select DEGs on the results. [file 1471-2105-11-S11-S5-S2.pdf]

**Table 1:** Number of correctly identified TFs by our method and Model I at different criteria on Hu et al. data.

| | Top 15 | Top 20 | Top 25 | Top 30 | Top 35 | Top 40 |
|---|---|---|---|---|---|---|
| Model I | 30 | 31 | 31 | 32 | 32 | 32 |
| Our method | 33 | 36 | 37 | 38 | 38 | 38 |

**Table 2:** Number of correctly identified TFs by our method and Model I at different criteria on Chua et al. data.

| | Top 15 | Top 20 | Top 25 | Top 30 | Top 35 | Top 40 |
|---|---|---|---|---|---|---|
| Model I on knockout data | 11 | 11 | 11 | 11 | 11 | 11 |
| Our method on knockout data | 11 | 11 | 12 | 13 | 13 | 13 |
| Model I on overexpression data | 11 | 11 | 11 | 11 | 11 | 11 |
| Our method on overexpression data | 15 | 17 | 18 | 20 | 20 | 20 |

Note :'Top N' means that if the actual perturbed TF is a valid finding (p-value<=0.01)and rank at the top N list of inferred candidates, this TF is said to be correctly identified.

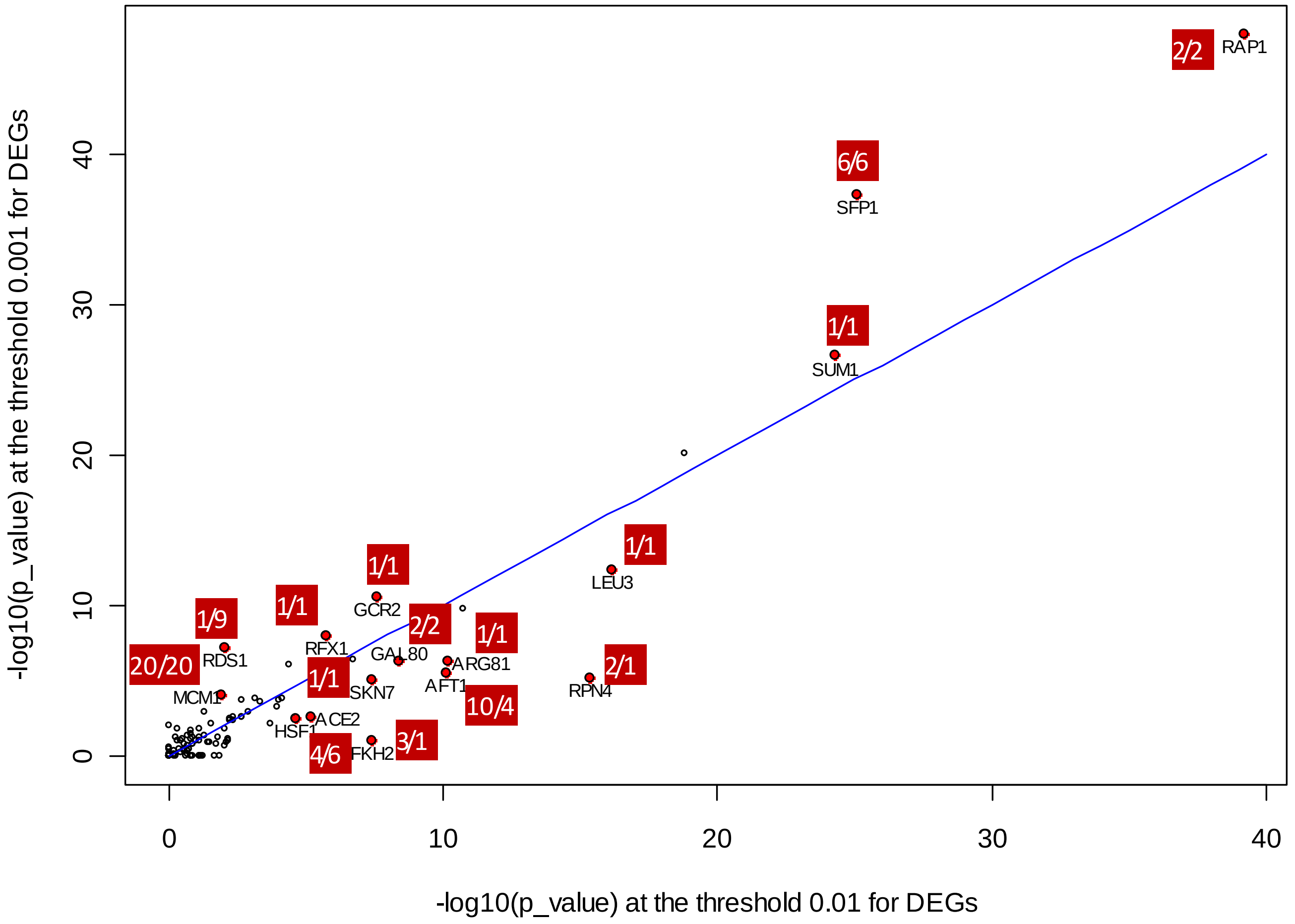


**Figure 1.** The overlap significance between expected targets and observed DEGs chosen at the threshold p<=0.01 (x axis) vs. that between expected targets and observed DEGs chosen at the threshold p<=0.001 (y axis) on Hu et al. data for 128 TFs. Red circles denote the difference between the overlap significance obtained at the threshold p<=0.01 and that obtained at p<=0.001 is larger than 100 times for those perturbed TFs. The number in the red rectangle denotes the rank of the perturbed TF in the list of candidates (the rank obtained at the threshold p<=0.001 for DEGs/ the rank obtained at the threshold p<=0.01 for DEGs). It could be seen that similar overlap p-values were obtained for most TFs even if different thresholds to select DEGs were used. Several TFs that got very different overlap p-value often ranked at the top of candidates, e.g., RAP1, SFP1, RPN4, FKH2, and RDS1. Furthermore, their ranks at the candidate list changed little though

their overlap p-values between expected targets and observed DEGs changed a lot. For example, although RAP1 got very different p-values when different thresholds were used to select DEGs (overlap p-value at the threshold 0.01 : 6e-40, p-value at the threshold 0.001: 1e-48), it ranked 2nd in both of these two conditions. As another example, although AFT1 got very different p-values at different thresholds, it ranked 4th when the threshold 0.01 was used and ranked 10th when 0.001 was used and the potential regulatory pathways downstream of AFT1 knockout were both PTM-mediated two-layer cascade regulation model.

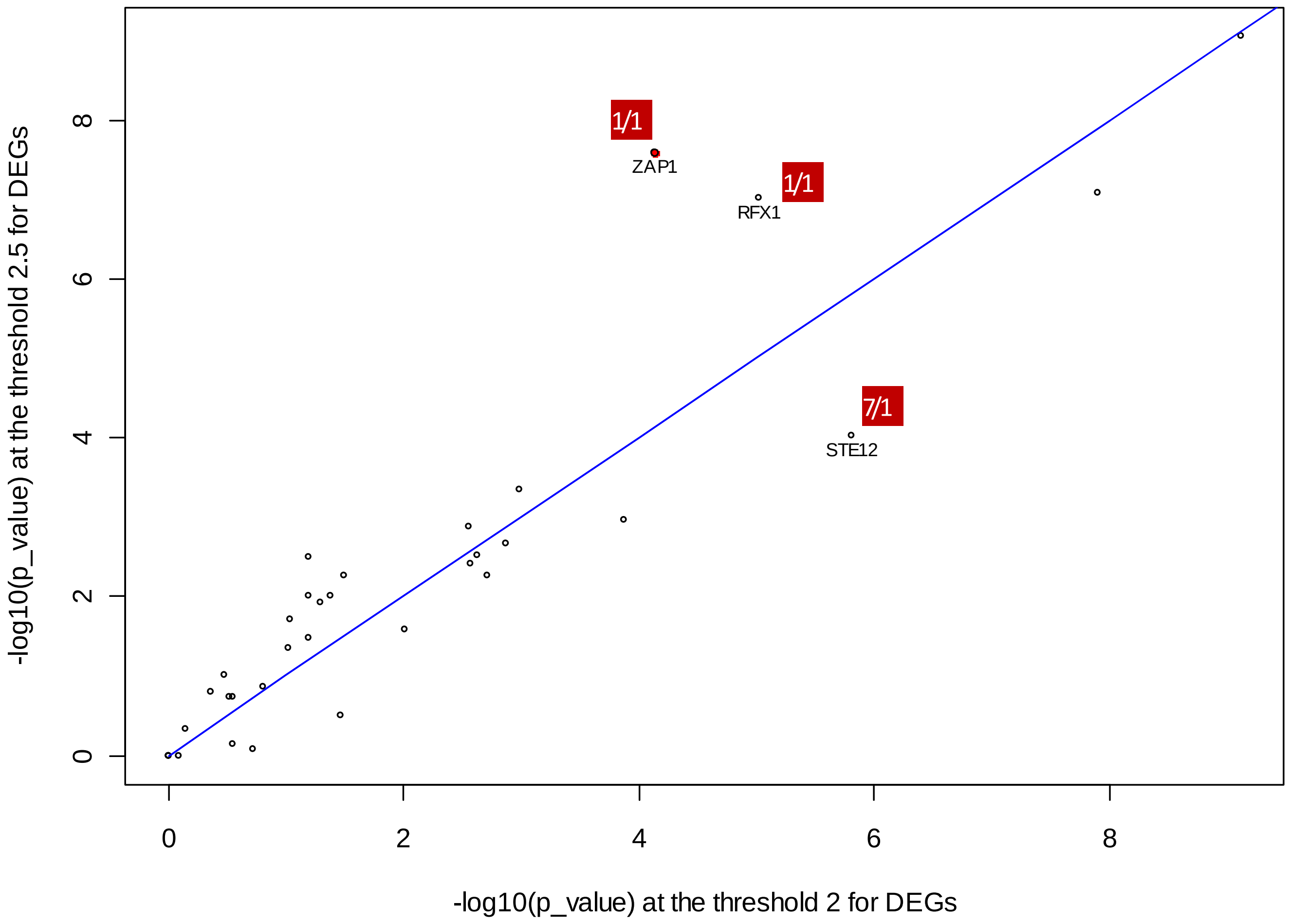


**Figure 2.** The overlap significance between expected targets and observed DEGs chosen at the threshold |z|>=2 (x axis) vs. that between expected targets and observed DEGs chosen at the threshold |z|>=2.5 (y axis) on Chua et al. knockout data for 35 TFs. Red circles denote the difference between the overlap significance obtained at the threshold |z|>=2 and that obtained at |z|>=2.5 is larger than 100 times for those perturbed TFs. The number in the red rectangle denotes the rank of the perturbed TF in the list of candidates (the rank obtained at the threshold |z|>=2.5 for DEGs/ the rank obtained at the threshold |z|>=2 for DEGs). It could be seen that similar overlap p-values were obtained for most TFs even if different thresholds to select DEGs were used. Only ZAP1 got very different p-value when different thresholds were used (the difference is larger than 100 times), but the rank of ZAP1 in the candidate list did not change at all (ranked 1st in both of these two conditions). RFX1 and STE12 also got different p-values (larger than 30 times), but their ranks did not change much.

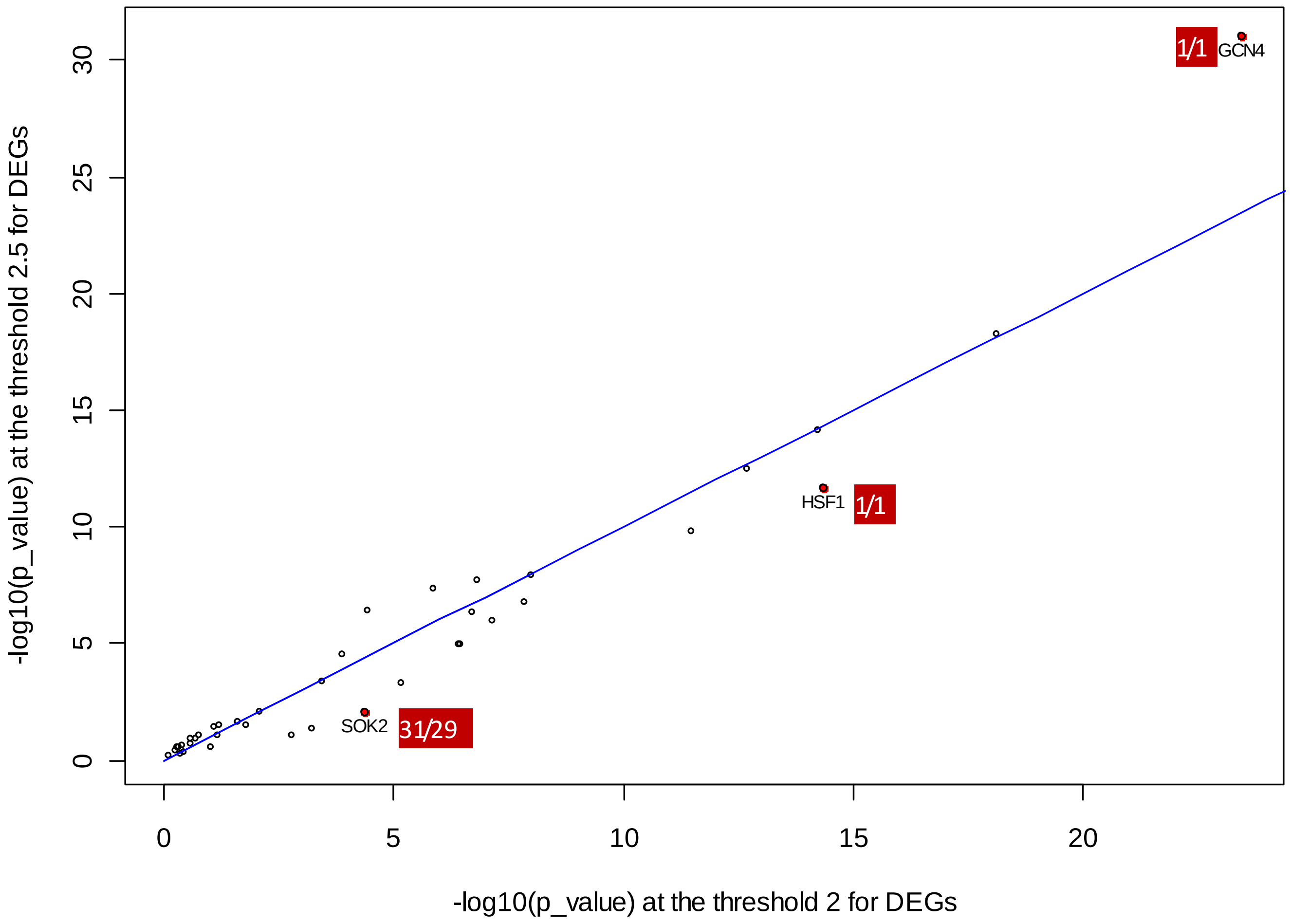


**Figure 3.** The overlap significance between expected targets and observed DEGs chosen at the threshold |z|>=2 (x axis) vs. that between expected targets and observed DEGs chosen at the threshold |z|>=2.5 (y axis) on Chua et al. overexpression data for 39 TFs. Red circles denote the difference between the overlap significance obtained at the threshold |z|>=2 and that obtained at |z|>=2.5 is larger than 100 times for those perturbed TFs. The number in the red rectangle denotes the rank of the perturbed TF in the list of candidates (the rank obtained at the threshold |z|>=2.5 for DEGs/ the rank obtained at the threshold |z|>=2 for DEGs). It could be seen that similar overlap p-values were obtained for most TFs even if different thresholds to select DEGs were used. GCN4, HSF1 and SOK2 got very different p-value when different thresholds were used (the difference is larger than 100 times), but the rank of these TFs in the candidate list did not change much at all.
