# Supplementary material for: TF-centered downstream gene set enrichment analysis: Inference of causal regulators by integrating TF-DNA interactions and protein post-translational modifications information
Source: BMC Bioinformatics. 2010 Dec 14;11(Suppl 11):S5. doi: 10.1186/1471-2105-11-S11-S5 (PMC3024863; doi:10.1186/1471-2105-11-S11-S5)
Supplement: Additional file 4 — Results on knockout data of 128 TFs. [file 1471-2105-11-S11-S5-S4.pdf]

Additional file 4 - results on knockout data of 128 TFs.

Available online at http://eng.scbit.org/pages/doc/supp_file_1.rar.

The supplementary includes 128 files. Each file contains the results on one perturbed TF experiment. For example, the file "YKL109W_HAP4" lists the results on the HAP4 knockout data. The number of downstream genes of TF in each model, the number of DEGs, the number of the overlap between downstream genes and DEGs, the overlap p-values obtained in each model, the minimum overlap p-value from the six models and the minimum p-value from acceptable models are listed in each file.
