# Supplementary material for: TF-centered downstream gene set enrichment analysis: Inference of causal regulators by integrating TF-DNA interactions and protein post-translational modifications information
Source: BMC Bioinformatics. 2010 Dec 14;11(Suppl 11):S5. doi: 10.1186/1471-2105-11-S11-S5 (PMC3024863; doi:10.1186/1471-2105-11-S11-S5)
Supplement: Additional file 5 — Results on deletion experiments of 35 TFs and overexpression ones of 39 TFs. [file 1471-2105-11-S11-S5-S5.pdf]

Additional file 5 - results on deletion experiments of 35 TFs and overexpression ones of 39 TFs.

Available online at: http://eng.scbit.org/pages/doc/supp_file_2.rar.

The supplementary includes 35 files for deletion experiments and 39 files for overexpression experiments. Each file contains the results on one perturbed TF experiment. For example, the file "HAP4.D" lists the results on the HAP4 deletion data and "HAP4OE" lists the results on the HAP4 overexpression data. The number of downstream genes of TF in each model, the number of DEGs, the number of the overlap between downstream genes and DEGs, the overlap p-values obtained in each model, the minimum overlap p-value from the six models and the minimum p-value from acceptable models are listed in each file.
Additional file 6- results on the expression profiles in the transition from fermentative to glycerol-based respiratory growth.
TFs are ranked by their minimum overlap p-values. The number of downstream genes of TF in each model, the number of DEGs, the number of the overlap between downstream genes and DEGs, the overlap p-values obtained in each model, and the minimum p-value are listed in the file.
